# Harnessing the Stem Cell Niche in Regenerative Medicine: Innovative Avenue to Combat Neurodegenerative Diseases

**DOI:** 10.3390/ijms25020993

**Published:** 2024-01-12

**Authors:** Gordana Velikic, Dusan M. Maric, Dusica L. Maric, Gordana Supic, Miljan Puletic, Oliver Dulic, Danilo Vojvodic

**Affiliations:** 1 Department for Research and Development, Clinic Orto MD-Parks Dr. Dragi Hospital, 21000 Novi Sad, Serbia; 2 Hajim School of Engineering, University of Rochester, Rochester, NY 14627, USA; 3 Faculty of Stomatology Pancevo, University Business Academy, 26000 Pancevo, Serbia; miljenko.puletic@gmail.com; 4 Department of Anatomy, Faculty of Medicine, University of Novi Sad, 21000 Novi Sad, Serbia; 5 Institute for Medical Research, Military Medical Academy, 11000 Belgrade, Serbia; gogasupic@gmail.com (G.S.); vojvodic.danilo@gmail.com (D.V.); 6 Medical Faculty of Military Medical Academy, University of Defense, 11000 Belgrade, Serbia; 7 Department of Surgery, Faculty of Medicine, University of Novi Sad, 21000 Novi Sad, Serbia; oliver.dulic@mf.uns.ac.rs

**Keywords:** stem cell niche, neurodegenerative diseases, Alzheimer’s disease, Parkinson’s disease, organoids, tumorigenesis, neurogenesis, morphogenetic fields, self-regeneration, artificial intelligence

## Abstract

Regenerative medicine harnesses the body’s innate capacity for self-repair to restore malfunctioning tissues and organs. Stem cell therapies represent a key regenerative strategy, but to effectively harness their potential necessitates a nuanced understanding of the stem cell niche. This specialized microenvironment regulates critical stem cell behaviors including quiescence, activation, differentiation, and homing. Emerging research reveals that dysfunction within endogenous neural stem cell niches contributes to neurodegenerative pathologies and impedes regeneration. Strategies such as modifying signaling pathways, or epigenetic interventions to restore niche homeostasis and signaling, hold promise for revitalizing neurogenesis and neural repair in diseases like Alzheimer’s and Parkinson’s. Comparative studies of highly regenerative species provide evolutionary clues into niche-mediated renewal mechanisms. Leveraging endogenous bioelectric cues and crosstalk between gut, brain, and vascular niches further illuminates promising therapeutic opportunities. Emerging techniques like single-cell transcriptomics, organoids, microfluidics, artificial intelligence, in silico modeling, and transdifferentiation will continue to unravel niche complexity. By providing a comprehensive synthesis integrating diverse views on niche components, developmental transitions, and dynamics, this review unveils new layers of complexity integral to niche behavior and function, which unveil novel prospects to modulate niche function and provide revolutionary treatments for neurodegenerative diseases.

## 1. Introduction

Regenerative medicine is a rapidly evolving, interdisciplinary field that combines expertise from genetics, biology, chemistry, general medicine, robotics, and computer science to explore the rejuvenation and repair of malfunctioning tissues and organs. Unlike traditional medical approaches that may address dysfunction through tissue removal or alternative treatments such as pacemaker implants or insulin prescriptions, regenerative medicine seeks to leverage the body’s intrinsic ability to heal and restore itself.

The techniques in regenerative medicine include stem cell therapies, which involve injecting stem or progenitor cells to enhance our natural healing capabilities. Although the term “regenerative” is often closely associated with stem cell use, it is merely one of several strategies employed in this domain. Nevertheless, stem-cell-based interventions hold considerable promise. Yet, to effectively harness their potential, it is crucial to grasp the intricate mechanisms that underlie regenerative processes.

Recent research has challenged conventional beliefs, revealing that human regenerative capacities may be dormant or obscured, rather than entirely diminished or absent. For example, historically, the prevailing belief was that human brain regeneration was either nonexistent or minimal. This view has evolved since due to new insights into stem cell mechanisms and their origins, supported by documented cases of unexpected recoveries [1,2,3]. Additionally, in the quest to unlock regenerative mechanisms, researchers have focused on organisms with remarkable regenerative capabilities, some of which can fully regenerate their brains.

While stem cells have received significant attention, the manipulation of stem cell niches to boost regeneration has been overshadowed. Emerging research emphasizes the critical role of surrounding factors in the success of cell therapies. Aspects such as signaling pathways, cell fate, and mobilization are influenced by the surrounding physical, biological, and chemical environment. This indicates that stem cell niches are integral to effective stem cell function. Therefore, targeting and rejuvenating compromised stem cell niches offers a potential pathway to mitigate, or even reverse, the harmful effects of neurodegenerative diseases.

The precise boundaries and locations of these niches remain enigmatic and require further exploration. Their locations could unveil unexpected relationships and pathways. For instance, these niches might be linked to neural pathways that stimulate organs like the heart and gut, thereby integrating them into the broader signaling network of the gut–brain axis and vascular connections [4].

Neurodegenerative diseases, including Alzheimer’s, Parkinson’s, and Huntington’s, are characterized by the progressive loss of neuronal structure and function. Recent studies suggest an intriguing link between these diseases and aberrations within neural stem cell niches. For instance, alterations in the subventricular zone (SVZ) and the subgranular zone (SGZ) of the hippocampal dentate gyrus (DG)—both critical for adult neurogenesis—have been observed in Alzheimer’s disease (AD) patients, indicating a disruption in niche homeostasis [5]. Such disruptions could compromise neuroplasticity and cognitive function, exacerbating the pathological features of these diseases. Cutting-edge research on epigenetic modifiers and niche signaling pathways is unveiling deeper layers of complexity. Investigations into, but not limited to, Wnt, Notch, and bone morphogenetic protein (BMP) signaling cascades, are revealing their convoluted roles in niche–stem cell crosstalk and neurodegenerative pathology [6,7,8].

The intricate relationship between stem cell niches and regenerative medicine holds significant implications for the treatment of neurodegenerative diseases. The reasons are twofold. First, the potential role of adult neurogenesis in neurodegenerative diseases offers avenues for understanding disease mechanisms. Second, the prospect of regenerative medicine opens the door for innovative therapeutic interventions. Regenerative therapies hold tremendous potential for restoring structure and function in neurodegenerative diseases like AD and Parkinson’s disease (PD) that currently lack effective treatments (Table 1). This review article aimed to analyze how elucidating and targeting endogenous stem cell niches may enable the development of innovative regenerative strategies to combat neurodegeneration. To do so, we provided a holistic synthesis of neural stem cell niche research, incorporating diverse elements like immune regulation, biomaterials, morphogen gradients, and some unconventional and modern approaches, or comparative regeneration studies, to reveal unappreciated connections and opportunities to modulate niche behavior for neurodegenerative diseases. The diverse perspectives on stem cell niches presented in this article showcase the niche’s multidimensional complex roles in regulating endogenous and transplanted stem cells. Integrating these views promises more nuanced understanding of niche complexity, illuminating new therapeutic avenues to harness humans’ latent regenerative potential.

## 2. Stem Cell Niches: Important Postulations

### 2.1. Definition

The stem-cell niche is a dynamic microenvironment in which stem cells maintain their viability and fulfill the stemness as a response to the distress and other signaling received from the macroenvironment. It is a complex term that denotes the anatomy of the physical location and its chemical, biological, and physical properties that govern the processes which enable the stem cells to fulfill their role. Although a unique definition of the stem-cell niche has yet to be established, it is explicit that the niche is essential for the proper maintenance and deployment of stem cells.

### 2.2. Stemness and the Niche

**Figure 1:**
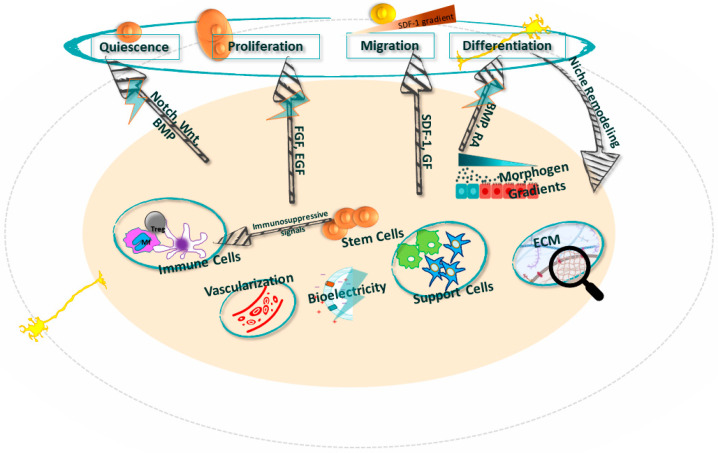
Mechanisms behind the stemness of the niche. For the sake of simplicity, the arrows show basic signaling pathways that influence different stemness functions. See Figure 2 for icon details.

Stemness comprises the unique properties of stem cells, i.e., everything that makes a stem cell the stem cell. For the lack of a unique definition of stemness, we will define stemness as the ability of stem cells to replicate, differentiate, and have a balanced interaction with the environment in terms of their dormancy, proliferation, mobilization, differentiation, homing, and regeneration. From that aspect, the niche is a supportive structure that enables stemness (Table 2, Figure 1). From a regenerative perspective, it is crucial to understand the influence of the niche on transforming the stem cell into the desired cell, followed by proper homing to the place of distress. The main puzzle is whether the niches are enablers or designers of the cell fate. The evidence that niches may be a source of pathology by changing the regular function of the stem cell or that artificial niches for in vitro culturing of stem cells may result in stem cell aging, a process which violates their stemness, points to the high importance of understanding the role of the niche in directing the regenerative flow. From the stem cell perspective, the niche is a cell’s playground, which highlights the activities of stem cell mobilization and their integration into functional tissues.

### 2.3. Elements

**Figure 2:**
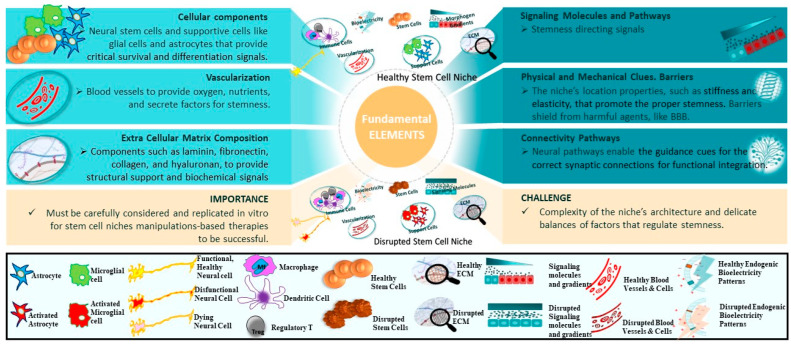
Key elements of the niche. The upper and lower plots in the middle depict healthy and diseased niches, respectively. The legend strip at the bottom illustrates the stages of the elements included in the middle plots. The text boxes on the sides list fundamental elements and the importance and challenge of the niches’ elements’ research and deployments.

The niche’s elements collectively govern the behavior of neural stem cells through a complex and dynamic interaction, especially within the context of neurodegenerative diseases [24]. The main elements of a niche include cellular components, signaling molecules, extracellular matrix (ECM), blood vessels, and other factors that influence the stemness in the niche (Figure 1 and Figure 2).

#### 2.3.1. Cellular Components

*Neural stem cells (NSCs)*. Primarily located in the SVZ and DG, NSCs are critical for maintaining neurogenesis and neural repair capacity in the adult mammalian brain. They activate in response to injury and diseases, and they maintain neurogenic activity through adulthood [16]. NSCs generate transit-amplifying progenitors that differentiate into neurons, astrocytes, and oligodendrocytes throughout life, and the rate of this process decreases with age. Their stemness is tightly regulated by supporting niche cells and signaling factors to match tissue demands and prevent stem cell exhaustion. In contexts of injury, disease, or aging, NSCs respond by proliferating and mobilizing to sites of damage in attempts to enact repair. Alterations in this environment often correlate with the pathophysiology of neurodegenerative diseases [24]. For example, chronic neurodegenerative conditions like AD and PD correlate with pathological microenvironments that disrupt the NSC niche, ultimately depleting the NSC pool and impairing neuroregenerative capacity over time.

*Supporting Cells*. Beyond NSCs, other critical cellular components populate neurogenic niches and regulate stemness, especially under pathological conditions like neurodegenerative diseases [25]. Astrocytes are specialized glial cells that nurture neural stem cells by secreting soluble factors like Wnts, BMPs, and Sonic hedgehog protein (Shh) that stimulate proliferation and fate specification [26]. Astrocytes also modulate synaptic transmission and immune activity, which become particularly relevant in contexts of inflammation-driven neurodegeneration [27].

Microglia similarly secrete pro- and anti-inflammatory signals influencing NSC quiescence, activation, and survival [28]. Microglial dysfunction and chronic neuroinflammation are implicated across multiple neurodegenerative diseases, necessitating tighter regulation. Finally, vascular endothelial cells modulate neural stem cell behavior through contact-mediated signals and secreted factors like brain-derived neurotrophic factor (BDNF) and vascular endothelial growth factor (VEGF) [29], which decrease in aging and disease. Dynamic interactions between these niche cells and NSCs may uncover new therapeutic roads for combating neurodegeneration.

#### 2.3.2. Signaling Molecules

Cellular components provide the structural and functional backdrop, while the specific signaling molecules in the niche further modulate stem cell behavior.

*Neurotrophins and Growth Factors.* Neurotrophins and growth factors are critical NSC niche regulators, supporting stem cell maintenance, survival, and differentiation.

BDNF, nerve growth factor (NGF), and *VEGF* are key molecules that maintain the survival, proliferation, and differentiation of NSCs [30]. Alterations in these signaling pathways are often implicated in neurodegenerative diseases [15,31,32,33]. Neurotransmitters have also been implicated in regulating neural stem cell behavior in the brain. For example, gamma-aminobutyric acid (GABA), a major inhibitory neurotransmitter, has been shown to regulate the proliferation of neural stem cells in the SVZ. GABAergic signaling influences the balance between quiescence and activation of neural stem cells [34].

*Cytokines and Chemokines.* These signaling molecules serve dual roles in the niche functioning environment, either maintaining NSC quiescence in the healthy niche or promoting NSC activation and differentiation in response to injury or disease. The chemokine CXCL12, also known as stromal cell-derived factor 1 (SDF-1), and its signaling receptor CXCR4, represent an important pathway that regulates the homing and maintenance of NSCs in neurogenic niches, such as in the SVZ [35]. Within these specialized microdomains, CXCL12 is secreted by endothelial and ependymal cells to communicate with NSCs expressing CXCR4, influencing both NSC quiescence and activation.

Neuroinflammation, a biologically complex response to damaging stimuli driven by proinflammatory cytokines and chemokines, is increasingly recognized as a key contributor to neuronal injury and neurodegenerative diseases [36]. Chemokines and cytokines modulate intricate neuroinflammatory responses by stimulating immune cell recruitment, activation, and proliferation [37,38]. Both pro- and anti-inflammatory mediators play crucial balancing roles—dysregulation of these responses can lead to chronic inflammation and associated pathologies. For instance, the proinflammatory cytokines interleukin-1 (IL-1), IL-6, and tumor necrosis factor-alpha (TNF-α) are elevated in many neurodegenerative states. They can stimulate damaging reactive astrocytes and microglia, compromise blood–brain barrier (BBB) integrity, and directly injure neurons and oligodendrocytes to drive disease progression. In contrast, anti-inflammatory factors like IL-4, IL-10, and transforming growth factor-beta (TGF-β) antagonize these effects and help resolve inflammatory responses. The factors tipping this balance towards chronic, damaging inflammation in neurodegenerative disease are multifaceted. Genetic polymorphisms, age-related immune senescence, and environmental stimuli can all dysregulate cytokine and chemokine production and immune cell function [39,40]. Additional studies clarifying these intricate signaling networks may uncover new therapeutic targets for resolving neuroinflammation and impeding further neuronal injury in neurodegenerative diseases.

#### 2.3.3. Extracellular Matrix

The ECM forms a complex network of proteins and polysaccharides, providing essential structural and biochemical support for maintaining tissue integrity. The intricate composition and organization of the ECM collectively shape the micro-environment, offering the necessary cues to sustain stemness within the specialized niche. In adult stem cell niches, the ECM directly engages with stem cells through cell adhesion receptors and indirect modification of growth factors and cytokines [41]. These biochemical and mechanical cues from the specialized ECM microenvironment are critical for regulating stemness. In neurogenic niches like the SVZ, NSCs interact with ECM proteins like laminin that promote stem cell renewal and maintenance. The ECM not only serves as a storage for morphogens like Wnts, but also modulates their activities. These Wnts are recognized as proneurogenic factors that play a crucial role in driving the process of neuronal differentiation [12]. Alterations to the ECM influence the proliferative capacity of NSCs in the aging brain [5]. In neurodegenerative diseases, abnormal accumulation or depletion of specific ECM proteins occurs, disrupting the stem cell niche microenvironment. For example, perineuronal net degradation around parvalbumin interneurons precedes gamma oscillation deficits in AD models [42]. Targeting the complex interactions between neural stem cells and the surrounding ECM niche represents an emerging avenue for brain repair strategies.

#### 2.3.4. Blood Vessels

Blood vessels are critical structural and functional components of adult stem cell niches, including neurogenic niches in the brain [1]. They modulate oxygen levels, provide nutrients, remove waste, and deliver systemic signals that regulate stemness [24,43,44]. In the adult mammalian brain, NSCs reside in vascular niches such as the lateral ventricles’ highly perfused SVZ and the hippocampus’s DG [45]. Here, NSCs directly interact with endothelial cells and pericytes that comprise the vasculature. These niche cells secrete soluble factors like BDNF, VEGF, and CXCL12 that support NSC functions.

Beyond nurturing niche homeostasis, vascular cells also couple systemic cues to dynamic modulation of neurogenesis and have emerged as critical players in NSC activation and mobilization after brain injury [6]. Damage to the vasculature strongly compromises neurogenic capacity and can contribute to neurodegenerative diseases. Preserving vascular health and angiogenesis is thus essential for maintaining NSC pools and regenerative potential. Targeting shared pathological mechanisms of vascular dysfunction and neurodegeneration represents a promising therapeutic approach.

#### 2.3.5. Other Factors

Stem cell behavior is not solely dictated by chemical signals. Beyond signaling molecules, interaction with the physical architecture and support systems, like the ECM and blood vessels, offers another layer of regulation and influence. Physical forces, such as those exerted by blood flow or by neighboring cells pushing against a stem cell, also affect their behavior. For instance, in the bone marrow, the sheer force exerted by blood flow can impact hematopoietic stem cell function. Certain stem cell niches, such as those within the bone marrow, thrive in environments with lower oxygen levels. This hypoxic setting plays a pivotal role in preserving stem cells in their undifferentiated state, preserving their unique potential and providing a foundation for the hyperbaric treatment in regenerative medicine [11,46,47,48]. In addition, niches may shield stem cells from toxins or harmful agents. Over time, alterations in the niche environment, often due to the aging process, disease, or injury, can exert a profound influence on how stem cells behave and execute their functions [3,24,49,50,51,52,53].

### 2.4. Location and Dynamics

The stem cell niche is not an anatomical formation in the traditional sense, like an organ or a specific tissue structure. Rather, it is a specialized microenvironment where stem cells reside, and it consists of various cellular and noncellular components that interact in complex ways to regulate stem cell behavior. The term “niche” in this context is more conceptual, describing a set of conditions and interactions that govern the fate of stem cells. Stem cell niches are often localized within specific anatomical regions. For example, in the mammalian brain, neurogenic niches are primarily found in the SVZ of the lateral ventricles and the SGZ of the hippocampal DG; intriguingly, these regions mediate learning/memory and olfaction, faculties prominently impacted in neurodegeneration. However, evidence exists that they may be forming in other areas [31,44,54,55].

Niche components are not static. Aging, injury, disease, and developmental stage can all alter cellular and molecular niche composition, impacting stemness [3,56]. For example, during development, the mammalian neurogenic niche disappears from most brain regions, persisting only in selective domains, like SVZ and SGZ [11]. Beyond compositional changes, stem cells can migrate to and from niches. This homing ability allows, for example, HSCs to circulate and then return to the bone marrow [57,58]. Under certain conditions, entirely new stem-like niches may emerge, like gliosis-mediated progenitor niches in brain lesions [59]. While anchoring stem cells spatially, the niche dynamically adapts its composition, function, and location to meet changing demands over an organism’s lifetime. Ongoing studies of niche plasticity promise deeper insight into how these microenvironments regulate stem cells under diverse physiological states.

## 3. The Multidimensional Landscape of Stem Cell Niches: Interplay, Influences, and Implications Inside and Out

**Figure 3:**
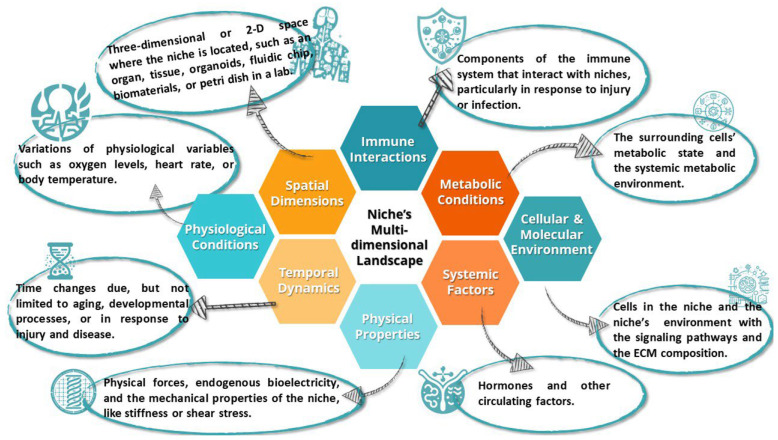
Basic elements of niche’s multidimensional landscape.

The niche’s multidimensional landscape opens the complex and dynamic set of conditions, factors, and interactions that characterize the niche. A fine-tuning of the landscape’s entities’ interactions may draw a difference line between a healthy and disrupted niche (see Figure 3).

### 3.1. The Dual Role of Neural Stem Cell Niches: Sustaining Neurogenesis and Contributing to Neurodegenerative Pathologies

Neurodegenerative diseases are characterized by the progressive degeneration of neurons and neural networks. An important yet often overlooked aspect of understanding these diseases lies in exploring the NSC environments that contribute to neural maintenance and repair.

The specialized microenvironments, also known as stem cell niches, play an integral role in maintaining and regulating NSCs in the adult mammalian brain. Key niches harboring NSCs include the SVZ of the lateral ventricles and the SGZ of the DG [24]. NSCs in these regions can give rise to new neurons, astrocytes, and oligodendrocytes throughout life through the process of neurogenesis. However, this endogenous neurogenic capacity appears to decline or become dysregulated in the context of neurodegenerative diseases like AD and PD [32].

In AD, beta-amyloid plaques and neurofibrillary tangles accumulate in the brain parenchyma surrounding NSC niches. Numerous studies in transgenic AD mouse models have shown reductions in hippocampal neurogenesis, which may be attributed to niche alterations including aberrant Wnt signaling, neuroinflammation, vascular deficits, and hypometabolism [33]. The Wnt pathway is critical for adult neurogenesis, but its activity is decreased in AD due to factors like amyloid-beta aggregation [15]. Specifically, beta-amyloid increases secretion of Dickkopf-1, which is antagonist of the Wnt receptor, to potentially disrupt Wnt/β-catenin signaling, which is essential for NSC proliferation and differentiation [60]. Neuroinflammation mediated by reactive astrocytes and microglia and their secreted proinflammatory cytokines can also lead to impaired NSC function and stunted neuronal maturation [61]. Key inflammatory cytokines shown to inhibit hippocampal neurogenesis in AD models include interleukin (IL) 1β (IL-1β), IL-6, TNF-α, and TGF-β [36]. Cerebrovascular changes like cerebral amyloid angiopathy that diminish blood flow to neurogenic niches likewise appear to contribute to dampened neurogenesis in AD [50]. Metabolic deficits and insulin resistance evident in AD may also negatively impact neurogenesis by disrupting energy metabolism, growth factor signaling, and gene regulation in NSCs [51].

Similar niche perturbations have been observed in the SVZ in PD, where α-synuclein (α-syn) pathology and neuroinflammation disrupt neuroblast migration to the olfactory bulb [44]. PD neuropathology in the SVZ stem cell niche alters FGF-2 signaling and neurotrophic factor levels like glial-cell-derived neurotrophic factor (GDNF), BDNF, and VEGF, creating a nonconductive environment for continued neurogenesis [62]. The accumulation of α-syn also elicits microglial activation and the release of inflammatory molecules like IL-1β, TNF-α, iNOS, and COX-2, which impair NSC viability and neuronal differentiation [63].

While intrinsic NSC defects may also contribute, niche-mediated mechanisms likely play a major role in hampering endogenous brain repair. Strategies to reverse niche dysfunction hold promise for stimulating regeneration in neurodegenerative disease [49]. For example, young blood transfusion, anti-inflammatory agents, exercise, dietary restriction, and pharmacological compounds have shown a preliminary potential to revitalize NSC niches in preclinical models [64]. Plasma from young mice contains factors like GDF11 that can enhance SVZ neurogenesis in aged mice, suggesting a rejuvenating effect [65]. Drugs that reduce the amyloid burden or modulate Wnt, BMP, Notch, and other signaling pathways could also restore neurogenic niche function in AD models [66]. Targeting the vascular component via statins, ACE inhibitors, or VEGF administration may promote neurogenesis by improving niche perfusion [64].

However, challenges remain in enhancing the integration and survival of newborn neurons in a diseased brain milieu. The pro-inflammatory environment, buildup of toxic protein aggregates, and lack of trophic support counteract the benefits of increased neurogenesis [67]. For truly effective brain repair, both the niche-based induction of new neurons and their supportive integration must be achieved. Developing multi-pronged approaches that combine neurogenesis-stimulating compounds with anti-inflammatory, anti-aggregation, and neurotrophic agents may help surmount this difficulty. Optimization of treatment timing and dosage is also critical, as factors like GDNF have shown dichotomous effects on neurogenesis depending on the disease stage [68].

### 3.2. Sophisticated Balance between Immune System and Stem Cell Niche in Neurodegenerative Diseases

The interplay between the immune system and the neural stem cell (NSC) niche is of paramount importance for maintaining brain health. This sophisticated balance serves as a double-edged sword, capable of both fostering and undermining neurogenesis.

Immune cells can exert a multi-faceted beneficial impact on stem cell behavior and the dynamics of their niches, ranging from direct cellular support to broader roles in maintaining a conducive biochemical environment. However, they can have detrimental influences. Macrophages stand as a prime example of immune cells that can exert beneficial and detrimental influences on NSCs as supportive mediators to NSC proliferation and differentiation. Macrophages achieve this by releasing growth factors and anti- and pro-inflammatory cytokines that create a conducive or inhibitory environment for NSCs. Macrophages can have different effects on NSCs depending on their phenotype and the microenvironment of the CNS. For example, M1 macrophages can release pro-inflammatory cytokines, such as IL-1β, TNF-α, and IFN-γ, that can impair NSC viability and differentiation. M2 macrophages can release anti-inflammatory cytokines, such as IL-4, IL-10, and TGF-β, that can promote NSC survival and differentiation. M2 macrophages can also secrete growth factors, such as GDNF, BDNF, and VEGF, that can stimulate neurogenesis and angiogenesis [69].

The balance can be disrupted in the context of chronic inflammation, a hallmark of neurodegenerative diseases [70]. Abnormally activated microglia are thought to drive the pro-inflammatory changes in neurodegenerative disease that counteract the neurosupportive immune environment required for neurogenesis [71]. Pro-inflammatory cytokines released by microglia, such as IL-1β, IL-6, and TNF-α, can inhibit hippocampal neurogenesis at multiple stages from NSC proliferation to neuronal maturation [72]. Persistent inflammation alters signaling dynamics essential for the NSC niche, including dysregulation of BMP, Notch, and Wnt pathways [73]. Furthermore, inflammasome activation triggers the release of IL-1β and IL-18, exacerbating inflammatory-induced niche impairments and reduced neurogenesis [74].

Targeting immune dysfunction and restoring balanced niche–immune interactions represent a promising avenue for therapeutic development. Regulatory T cells help constrain CNS inflammation and have been shown to rescue age-related neurogenesis decline when their numbers were boosted in an animal model [75]. Additionally, blocking IL-1R signaling can ameliorate microglia-mediated suppression of neurogenesis [76]. The sophisticated crosstalk between the immune system and the stem cell niche wields tremendous influence over neurogenesis outcomes in the healthy and diseased brain. Future research should focus on elucidating mechanisms of neuroprotective vs. neurotoxic neuro-immune communication to uncover niche-modulating immunotherapies that stimulate regeneration in neurodegenerative disease. For instance, anti-inflammatory cytokines, such as IL-4 and IL-10, can induce an M2 phenotype in microglia and macrophages, which can promote NSC survival and differentiation. Growth factors, such as GDNF and BDNF, can stimulate the proliferation and maturation of new neurons, and protect them from inflammatory damage. Immunosuppressive drugs, such as cyclosporine A and rapamycin, can inhibit the activation of T cells and microglia, and enhance the function of regulatory T cells, which can reduce neuroinflammation and increase neurogenesis.

### 3.3. The Intricate Relationship between Natural Stem Cell Niches and Transplanted Stem Cells

Stem cell niches play a crucial role in housing and regulating endogenous stem cells in their native tissues. However, these niches also have significant implications for the field of regenerative medicine, serving as a blueprint for understanding how transplanted stem cells might behave in a new environment. There is an intricate relationship between natural niches and stem cell therapies that researchers are only beginning to unravel. In this section, we will discuss some of the key aspects of this relationship and how they can be exploited for therapeutic applications.

#### 3.3.1. Mimicking the Niche for Improved Engraftment

For exogenous stem cells to successfully engraft and function within a recipient, they often require an environment that closely resembles their native niche [77]. The niche provides a complex array of ECM components, cell adhesion molecules, growth factors, cytokines, and other signaling molecules that support stem cell residence and regulation. Recreating aspects of this niche environment, such as incorporating similar matrix proteins or pretreating cells with niche-derived regulatory factors, can greatly improve the ability of transplanted stem cells to survive and engraft [78]. For instance, neural stem cell grafts showed enhanced survival and integration when co-transplanted with vascular endothelial cells, which are key components of the neurogenic niche due to secretion of BDNF—a growth factor that supports neuronal survival and differentiation [79].

#### 3.3.2. Regulation of Stem Cell Survival, Proliferation, and Differentiation

Once transplanted, stem cells need the right signals to survive, expand, and differentiate appropriately for therapeutic purposes. The natural stem cell niche provides a suite of molecular factors, ECM components, and cellular interactions that tightly control these stem cell behaviors [80]. Growth factors like FGF, EGF, and VEGF promote stem cell division, while cell cycle inhibitors and differentiation factors like p21, p27, BMPs, and Notch ligands ensure controlled proliferation and specification [81]. Cell–cell and cell–matrix adhesion molecules also influence stem cell fate. Without a niche-mimicking environment, transplanted stem cells risk death, aberrant proliferation, or improper differentiation post-engraftment. For instance, neural stem cells transplanted without niche support default to an astrocytic fate rather than correctly producing neurons [82,83,84].

#### 3.3.3. Promoting Integration with Host Tissue

In addition to supporting stem cells, the niche facilitates seamless integration with the surrounding tissue. Successful transplantation requires an intricate exchange of signals between grafted cells and the host niche to enable appropriate tissue integration [48]. For example, vasculature-derived factors prompt neural stem cells to extend processes toward blood vessels during incorporation into the brain [9]. Adopting strategies to present key niche ECM molecules, growth factors, and cell adhesion ligands at the transplant site could enhance stem cell incorporation and synergy with endogenous cells.

#### 3.3.4. Avoiding Overgrowth and Tumorigenesis

A major risk of stem cell therapy is uncontrolled proliferation leading to teratoma or tumor formation. The natural niche tightly regulates stem cell division through symmetric vs. asymmetric cell division, quiescence signals, and other mechanisms [81]. Note that the mode of division determines whether stem cells produce two identical daughter cells or one stem cell and one differentiated cell. As one example, BMPs restrict neural stem cell expansion while promoting differentiation [85]. By reproducing such niche signals, transplanted stem cells can be induced to proliferate in a controlled, regulated manner.

#### 3.3.5. Modulating the Immune Response

Introducing exogenous cells can trigger a damaging immune reaction, leading to rejection of the transplanted stem cells. However, the native niche provides immune privilege via physical barriers, immunosuppressive factors, and a lack of MHC I expression on stem cells [86]. MHC I stands for major histocompatibility complex class I, a molecule that presents antigens to T cells and triggers an immune response. Harnessing such niche-derived immune-evading properties, such as delivering anti-inflammatory drugs or engineering stem cells to avoid immune detection, could help transplanted cells to escape rejection.

#### 3.3.6. Ex Vivo Niche Recreation

Certain advanced stem cell therapies require expanding or differentiating cells ex vivo before the transplantation. Ongoing research is focused on engineering artificial niches in the laboratory, equipping stem cells with vital niche factors needed for therapeutic applications prior to engraftment [87]. This could involve culturing cells on niche-mimicking ECM proteins, exposing them to key growth factors, genetic manipulation to enhance the stem cell’s expression of niche-responsive genes or receptors, or establishing co-cultures with niche-supportive stromal cell types.

#### 3.3.7. Understanding Stem Cell Homing Mechanisms

Some transplanted stem cells demonstrate remarkable homing capacity, migrating to sites of injury or disease [23,88]. The natural niche provides molecular guidance cues facilitating such directed stem cell motility [89]. For instance, SDF-1 chemokine signaling attracts circulating hematopoietic stem cells to the bone marrow niche [54,90]. Elucidating these homing signals and mechanisms offers opportunities to exploit stem cell homing for targeted, site-specific therapies.

The native stem cell niche has a profound influence over the behavior and functionality of transplanted stem cells. Further elucidating these complex interactions promises to unlock the full regenerative potential of stem cell therapies. Recreating key niche elements could support stem cell engraftment, integration, controlled expansion, and homing—catalyzing the advancement of regenerative medicine [10,91].

### 3.4. Engineering Artificial Niches through Biomaterials

Biomaterials are synthetic or natural materials that can interact with biological systems and modulate their function. Biomaterials offer a promising approach to recreate elements of the native stem cell niche for transplantation purposes, as they can provide physical, chemical, and biological cues to regulate stem cell behavior and tissue regeneration. By encapsulating stem cells in biomaterial scaffolds engineered to present niche-derived ECM proteins (such as collagen, laminin, and fibronectin), adhesion molecules (such as integrins, cadherins, and selectins), and signaling factors (such as growth factors, cytokines, and chemokines), engraftment and therapeutic outcomes can be significantly enhanced [92]. These biomolecules can influence stem cell survival, proliferation, migration, differentiation, and paracrine effects by activating specific receptors and signaling pathways on the cell surface. For example, neural stem cells displayed improved survival and differentiation when transplanted in hydrogel scaffolds containing laminin, an ECM protein found in neurogenic niches [93]. Controlled release of niche factors like BMP-2 from degradable microparticles embedded within cell scaffolds also promoted stem cell differentiation [94,95]. In addition to directly supporting transplanted cells, biomaterial scaffolds can be used to divert endogenous stem cells into injured areas. Injectable hydrogels delivering SDF-1 recruited endogenous neural progenitors when applied to stroke lesions in mouse models [96]. Biomaterials also show potential for recreating niche environments during ex vivo stem cell expansion prior to transplantation [97]. Biomimetic scaffolds provide a powerful platform for replicating native niche signals to direct stemness. However, there are still many challenges and limitations in using biomaterial scaffolds for stem-cell-based tissue engineering, such as optimizing the scaffold design and fabrication, ensuring biocompatibility and biodegradability, controlling the release kinetics and bioactivity of the encapsulated factors, mimicking the dynamic nature and complexity of the native niche, and evaluating the safety and efficacy of the scaffold–stem cell constructs in vivo.

### 3.5. Interactions with Induced Stem Cells

The reprogramming of adult somatic cells into induced pluripotent stem cells (iPSCs) stands as a landmark achievement in stem cell biology and regenerative medicine [98]. Researchers can differentiate iPSCs into neurons, astrocytes, oligodendrocytes, and microglia to investigate mechanisms of neuroprotection and neurorestoration. For example, iPSC-derived astrocytes might support motor neurons in amyotrophic lateral sclerosis (ALS)—a neurodegenerative disease that affects motor neurons—or replace dopaminergic neurons that produce dopamine, a neurotransmitter involved in movement control in PD [99,100]. To generate iPSCs in vitro, researchers often construct artificial stem cell niches by providing a milieu replete with growth factors, extracellular matrices, and cytokines that echo the endogenous niche environment. This bolsters iPSC derivation and ensures sustained self-renewal [101]. However, a latent risk remains—unbridled proliferation could culminate in teratoma formation, compromising the structural integrity of tissues post-transplantation [102]. Hematopoietic progenitors sourced from iPSCs might integrate into the bone marrow niches, thereby rivalling native hematopoietic stem cells [103]. This indicates that infusing derived cells into tissues can affect indigenous niches. In pathological states, maladaptive niche signals could destabilize iPSC-derived cells. This underscores the imperative for niche recalibration to ensure seamless integration and optimal cellular functionality [104].

Interestingly, transplanted iPSC derivatives attract ancillary cells, orchestrating novel microenvironments akin to transient stem cell niches [105]. The scientific community is also witnessing strides in biomaterial scaffolds and niche engineering modalities, tailored to shepherd the survival, integration, and modulated proliferation of iPSC-induced grafts [106].

## 4. The Power of the Stem Cell Niche in Advanced Regeneration

### 4.1. The Role of ACA in Stem Cell Reprogramming and Niche Modulation

The reprogramming of differentiated cells into iPSCs is primarily achieved through the introduction of specific transcription factors which reset the cells’ identity, making them revert to a pluripotent state that is similar to embryonic stem cells. The reprogramming efficiency can be influenced by various molecules and conditions. ACA (also known as CD133 or prominin-1) is a glycosylphosphatidylinositol (GPI)-anchored protein that is expressed on the surface of various stem and progenitor cells, including hematopoietic stem cells (HSCs), endothelial progenitor cells, neural stem cells, and cancer stem cells [107]. ACA emerges as a significant player in stem cell biology, and potentially within the context of the stem cell niche. ACA functions as an upstream regulator of human hematopoiesis, indicating that it might be influencing the behavior of HSCs within the niche, i.e., affecting how these cells respond to niche signals and how they proliferate and differentiate. In addition, ACA has the potential to induce pluripotency in blood progenitor cells without the need for genetic manipulation [108,109,110,111]. This is a groundbreaking revelation, as one of the significant concerns with induced pluripotent stem cell (iPSC) generation is the potential for genetic mutations and tumorigenesis. By utilizing ACA’s signaling pathways, it is possible to create a more “natural” pluripotent state stem cell niche environment in vitro, where cells revert to a pluripotent state in response to ACA signaling. The level of ACA receptor expression seems to determine the balance between pluripotent and differentiated states. This suggests that ACA might play a role in stem cell fate decisions within the niche, acting as a modulator that can “tune” stem cell behavior. A notable point is that ACA-induced pluripotent cells do not form teratomas. Teratoma formation is a significant risk with pluripotent cells, so this property of ACA-induced cells suggests that ACA might influence the niche in ways that suppress tumorigenesis. Considering these findings, ACA seems to play a multivariant role in stem cell niches. It acts as a regulator, a modulator of cell fate decisions, and potentially as a safety mechanism to prevent uncontrolled growth. It bridges the gap between the natural stem cell niche environment and the potential for therapeutic stem cell applications.

### 4.2. Endogenous Electrical Cues: Directing Stem Cell Dynamics and Tissue Repair

Bioelectric phenomena are endogenous electrical cues produced and sensed by cells that have emerged as vital regulators of various biological processes, including stem cell function, communication, and tissue regeneration. Endogenous bioelectric gradients are generated by ion channels, pumps, and transporters on the cell membrane that create differences in membrane potential and ion concentrations across cells and tissues. Endogenous bioelectric fields can dictate cell migration patterns, a phenomenon known as galvanotaxis or electrotaxis [112,113,114]. These fields can influence the movement and localization of stem cells within the niche or modulate their homing capabilities post-transplantation [115,116,117]. Within the niche, endogenous bioelectric gradients may serve as spatiotemporal guides for stem cell differentiation. The signals may bolster the brain’s inherent regenerative capabilities. Animals such as salamanders and zebrafish demonstrate the critical involvement of bioelectric signals in limb and organ regeneration by modulating the expression and activity of genes such as Hox, Msx, Pax, and Shh that are involved in patterning and morphogenesis [118,119]. Though not explicitly concerning stem cell niches, these regenerative phenomena often encompass niches that support stem or progenitor cells, offering vital clues into the role of bioelectricity in niche dynamics during tissue repair [120]. Cells within a niche can communicate through bioelectric signals, establishing interactive networks where electrical perturbations in one cell can affect neighboring cells [121]. Such bioelectric communication could synchronize behaviors among stem cells and their supportive niche components by creating gap junctions, which are intercellular channels that allow direct electrical coupling between adjacent cells. Post-injury, transient shifts in bioelectric properties often occur, colloquially termed as the “current of injury” [122], and diseases like AD alter neural network activities and bioelectric patterns by affecting the synaptic transmission, neuronal excitability, calcium signaling, and amyloid-beta production in neurons and glia [123,124]. These bioelectric alterations can cause inflammation and modulate the behavior of stem or progenitor cells in local niches, thereby potentially guiding tissue regeneration and repair or niche degradation. Specific voltage patterns could direct NSCs towards neuronal over glial differentiation by affecting the expression and activity of transcription factors such as NeuroD1, Neurogenin2, Mash1, Olig2, and Sox10 that are involved in neural lineage specification, particularly relevant in conditions where selective neuronal populations are compromised, or influence glial cell activity, implicating their role in modulating neuroinflammation prevalent in neurodegenerative diseases [125,126]. The exact voltage patterns that would promote neurogenesis over gliogenesis in neural stem cells are not well defined yet, although some studies suggest hyperpolarized membrane potentials favor neuronal differentiation, while depolarized potentials maintain multipotency or lead to glial fates [127,128]. Bioelectric cues affect the release and uptake of neurotransmitters such as glutamate, GABA, dopamine, serotonin, and acetylcholine that are fundamental for synaptic transmission and may influence stem cells within brain niches to maintain or restore synaptic integrity, often compromised in neurodegenerative conditions [129]. Understanding bioelectric cues could enable the enhancement of stem-cell-based therapies, direct tissue regeneration, and modulate endogenous stem cell behavior in pathological states. Techniques that apply weak electric currents to specific brain regions to modulate neuronal activity and plasticity, such as transcranial direct current stimulation (tDCS) and deep brain stimulation (DBS), might offer new avenues for modulating NSC niches and promoting neural repair [130,131]. Strategies like optogenetics, electromagnetic field exposure, and conductive biomaterials have been used to systematically modulate voltage in stem cells and investigate effects on lineage commitment by using light-sensitive ion channels, magnetic coils, or electrically conductive polymers or nanomaterials to manipulate membrane potential or ion fluxes in cells [132,133,134,135,136,137,138,139,140]. Bioelectric cues are essential regulators of stem cell niches in health and disease. They can dictate cell migration patterns, differentiation outcomes, communication networks, tissue integration, and homing capabilities. By understanding bioelectric cues, it is possible to enhance stem-cell-based therapies, direct tissue regeneration, and modulate endogenous stem cell behavior in pathological states. However, further elucidating the complex interactions between bioelectric signals and stem cells in different tissues and disease contexts is essential for optimizing bioelectric-based therapies. Moreover, developing novel methods to measure and manipulate bioelectric phenomena in vivo and ex vivo poses significant technical and ethical challenges. Therefore, advancing our understanding of bioelectricity and its implications for regenerative medicine requires multidisciplinary collaboration and innovation.

### 4.3. Morphogenetic Fields: Shaping Niche Dynamics and Stem Cell Behavior

The concept of morphogenetic fields originated in developmental biology to describe embryonic regions where cell fate specification occurs, often directed by diffusible morphogens forming gradients [141]. While initially framed in developmental contexts, the idea of morphogenetic fields may also provide insights into stem cell niche function and regulation.

Morphogen gradients could impose distinct cell fates based on concentration thresholds within niches, as with Shh patterning neural tube progenitors [142]. Niche morphogens may also help establish boundaries segregating stem cells from differentiating progeny, as retinoic acid does between hematopoietic stem cells and downstream lineages [143,144,145,146,147]. In regenerating systems like salamander limb regrowth, new morphogenetic fields are thought to guide tissue redevelopment, relying on stem cell pools to supply cells [148]. Feedback loops within niches could refine morphogen levels and stem cell outputs to enable regeneration. Beyond morphogens, biophysical forces generated during niche cell movements and interactions may influence behaviors like quiescence, activation, and egress [149]. As sensitive signaling centers, niches may link tissue-level morphogenetic fields to external cues from the broader environment. However, any aberration disrupting niche morphogen gradients or boundaries could impair stem cell function and tissue homeostasis. This is evident in squamous cell carcinomas, where disruption of morphogen signaling leads to dysplastic niche activity [150]. Nevertheless, niches may serve as key components interfacing with morphogenetic fields to regulate stem cell specification and tissue patterning. Further illuminating these dynamics could shed light on developmental and regenerative processes centered around maintaining stem cell populations.

### 4.4. Decoding Nature’s Masters of Regeneration: Insights into Niche Dynamics and Cellular Renewal

The basic genetic building blocks for regeneration are present in many species, including humans. However, the ways in which these genes are regulated, expressed, and the specific proteins they encode can vary significantly. Proteins, particularly those resulting from the expression of genes related to regeneration and development, play critical roles in the formation, maintenance, and regulation of the niches. The regeneration mechanisms reveal the complex mechanisms behind the regeneration capabilities. The complexity spans feedback mechanisms to ensure balance between stem cell maintenance and regeneration; the gradient of signaling molecules across the niche that dictates the behavioral phase of stem cells; serotonin accumulation at neural injury indicating involvement of neurotransmitters; the immune system which favors regeneration; spatial orientation, i.e., spatial cell memory; and neural signals which, for example, play a crucial role in limb regeneration in lizards. If the nerve is severed in the tail, regeneration is impaired [151,152,153]. In zebrafish heart regeneration, the ablation of epicardial niches housing muscle progenitors severely impair the regenerative response [154]. Planarians exhibit an extraordinary capacity to regenerate entire bodies from fragments of tissue. This relies on pluripotent adult stem cells called neoblasts that can give rise to all cell types. Post-injury, neoblasts migrate to wound sites and proliferate in response to conserved signaling pathways like Wnt, FGF, Hedgehog, TGF-beta, and Notch that regulate stem cell dynamics [155]. Axolotls are capable of regenerating complex structures like limbs, tails, jaws, and portions of the heart and brain. They activate progenitor-like cells called blastema at injury sites that orchestrate tissue redevelopment [156], while endogenous bioelectric signals guide the patterning of regenerating tissue, modulated through ion channels that control resting potential gradients [157]. In addition, axolotls, known for scar-free brain regeneration, activate unique glial cells in response to brain injury. The activated radial glial cells form a structure reminiscent of the neural stem cell niches seen during development [158,159,160,161]. Future research needs to elucidate spatiotemporal elements of animal regenerative mechanisms related to stemness and niches that can potentially open doors to evoke human regenerative potential on demand or per need.

### 4.5. Cross-Niche Collaborations and Systemic Influences

In the realm of regenerative medicine, exploring the intricate interplay between stem cell niches, the gut–brain axis, and vascular connections holds immense promise for understanding and potentially treating neurodegenerative diseases. Neurodegenerative diseases, such as AD and PD, exhibit complex pathological mechanisms that extend beyond the nervous system. The gut–brain axis is a complex network of communication between the gastrointestinal tract and the central nervous system, involving neural, endocrine, immune, and metabolic pathways. The gut–brain axis significantly influences disease progression in neurodegenerative diseases by modulating inflammation, oxidative stress, neurogenesis, and synaptic plasticity [3,162]. Stem cell niches within the gut and brain play pivotal roles in tissue maintenance and repair, but their dysfunction could contribute to disease [22,163]. The gut microbiota is the diverse community of microorganisms that inhabit the gastrointestinal tract and it produces various metabolites, neurotransmitters, and hormones that can cross the blood–brain barrier and influence neural function and behavior. For instance, some gut bacteria can produce short-chain fatty acids that modulate neuroinflammation and neurogenesis, or serotonin that regulates mood and cognition. Moreover, research on animal models has indicated that the gut microbiota can influence neural function and behavior, suggesting a potential link between the guts and brain stem cell niches and brain health. Notably, nanosized extracellular vesicles (EVs), including exosomes, have been identified within the SVZ, a region in the brain that contains neural stem cells that can generate new neurons and glia throughout life. The SVZ is one of the main stem cell niches in the brain, and its function and regulation are crucial for brain health and repair. This suggests the intriguing possibility that extracellular signals originating from distant sources could potentially reach the niche via cerebrospinal fluid (CSF) or the vasculature [164]. In addition, microbiome modulation may be a therapy target for niche modulation. Vascular impairment, a common feature in neurodegenerative disorders, further complicates the scenario by affecting nutrient supply and waste removal [165]. In addition, researchers identified a vagal afferent pathway that increases stomach–brain coupling [166], providing new insight into the neurobiological mechanisms underlying brain–gut communication and potential roles for neurotransmitters and the nervous system in interniche collaboration. By comprehensively examining these interconnected systems, we may unearth novel therapeutic avenues for neurodegenerative diseases, harnessing the potential of coupled niches and addressing systemic influences on disease manifestation.

### 4.6. Manipulating Epigenetic Signature in Stem Cell Niches and Its Implications for Neurodegenerative Diseases

Epigenetic modifications impact gene expression without changing the DNA sequence. Unlike static genetic changes, epigenetic changes are highly dynamic, potentially reversible and under the constant influence of environmental factors and cellular signals. Epigenetic memory is the phenomenon by which gametes can retain a record of their previous epigenetic modifications gained through life, exerting transgenerational epigenetic effects on gene expression in future generations.

The key epigenetic mechanisms include DNA methylation, histone modifications, chromatin remodeling, and noncoding RNAs. These mechanisms that add or remove methyl groups to the DNA, or various chemical groups to the histones (including methyl, acetyl, phosphate groups, etc.), alter the chromatin state and its accessibility to transcriptional machinery and interactions with transcription factors and other regulatory molecules [167]. Epigenetic mechanisms have been identified as important regulators in stem cell niches, serving as dynamic instruments for genes’ responses to intrinsic and environmental cues, such as oxygen levels, nutrient availability, mechanical stress, inflammation, hormones, and drugs. For instance, they can dictate whether a stem cell will remain quiescent, self-renew, or differentiate into a specific lineage for tissue repair [167]. A primary example is DNA methylation, which can lead to silencing genes essential for stem cell differentiation, thereby maintaining the stem cells in a pluripotent state [168]. Epigenetic alterations have been implicated in the pathogenesis of neurodegenerative diseases. In AD, epigenetic modifications such as DNA methylation and histone acetylation have been shown to influence the expression of genes which are key players in the amyloid-β pathway, a hallmark of the disease [169,170]. In Parkinson’s disease, epigenetic modifications affect the expression of α-syn, a protein that aggregates in the brains of the patients [171]. There is some evidence that epigenetics supports the idea that ACA influence is hidden in epigenetic memory. A study [108] showed that ACA-positive hematopoietic stem cells (HSCs) had higher levels of DNA methylation than ACA-negative HSCs, and that DNA methylation regulated the expression of genes related to HSC quiescence, self-renewal, and differentiation. This suggests that ACA might mark a subset of HSCs with a distinct epigenetic profile that confers on them stemness properties.

Epigenetics-based drugs may stimulate regenerative capacity in endogenous neural stem cells or enhance the integration and survival of exogenously derived cell transplants in the damaged niche. The histone deacetylase inhibitors sodium valproate, sodium butyrate, and vorinostat have been shown to enhance hippocampal neurogenesis from neural stem cells and improve cognitive function in mouse models of AD via targeted inhibition of class I histone deacetylases HDAC1, 2, 3, and 8, and the inhibition of HDAC6 by vorinostat [172]. Therefore, understanding the epigenetic influences on stem cell niches and targeting epigenetic mechanisms could potentially open new therapeutic avenues for neurodegenerative diseases. Modulating the epigenetic signatures critical to neural stem cell activity and reversing pathological epigenetic changes in the niche environment offer novel opportunities to regenerate neurogenic niches, reactivate endogenous regenerative pathways, and attenuate neurodegeneration.

### 4.7. Noncoding RNAs: Cell-Specific and Nonspecific Key Gene Regulators Implicated in Neurodegenerative Diseases

miRNAs and long noncoding RNAs (lncRNAs) are two families of noncoding RNAs (ncRNAs) that regulate gene expression at the post-transcriptional level. Their dysregulation has been implicated in diverse aspects of neurodegenerative pathology, including neuroinflammation, neuronal death, and protein aggregation [173].

miRNAs are essential post-transcriptional regulators of gene expression in the nervous system. Dysregulation of specific miRNAs contributes to the pathogenesis of diverse neurodegenerative diseases by targeting key mRNAs and signaling pathways involved in protein aggregation, neuronal differentiation, and survival, neuroinflammation, apoptosis, autophagy, and oxidative stress responses. For example, miR-149, miR-29, miR-15b, miR-125b, and miR-34 show altered expression across cellular and animal models of AD [174]. Downregulation of miR-29 enhances cell death programs in models of ALS [175]. Overexpression of miR-34 exacerbates α-syn accumulation in Parkinson’s disease models [176]. The emerging data reveal several overlapping miRs upregulated across the multiple neurodegenerative diseases, including miR-146a, miR-155, and miR-132 [177]. MicroRNA-based therapeutics shows promising potential in the mitigation of neural inflammation and improving outcomes in animal models [174]. Beyond intracellular functions, miRNAs can also be actively secreted in extracellular vesicles or exosomes, enabling cell–cell signaling within the neural niche microenvironment. These exosomal miRNAs represent promising biomarkers of neurodegenerative disease states and potential therapeutic candidates to combat inflammation, protein toxicity, and neural damage associated with neurodegenerative diseases.

miRNAs and lncRNAs play important roles in regulating microglial activation and polarization, the key processes underlying neuroinflammation. Microglia are the brain’s resident macrophages and play an important role in immune surveillance and tissue repair. Microglia can be polarized into two distinct phenotypes: M1 and M2. M1 microglia are pro-inflammatory and can contribute to neurodegeneration, while M2 microglia are anti-inflammatory and can promote neuroprotection by releasing trophic factors. The imbalance of these phenotypes contributes to chronic neuroinflammation in neurodegenerative disorders. Both miRNAs and lncRNAs have been shown to regulate the M1/M2 polarization of microglia. Specific miRNAs promote M1 polarization. miR-124 promotes neuronal survival and M2 polarization of microglia [178], while miR-128 promotes the microglia viability through downregulation of M1 polarization, upregulation of the M2 phenotype, and repression of inflammatory cytokine production [179]. Manipulating phenotype-driving miRNAs represents a viable approach to dampen chronic neurotoxic inflammation. Proper neuronal production from NSC niches like the ventricular-SVZ (V-SVZ) is essential for structural plasticity and function but deteriorates with aging. Various lncRNAs influence NSCs’ fate choices by interacting with intermediates in cell signaling pathways that direct self-renewal versus lineage commitment. The lncRNA Pnky is a key regulator of NSC differentiation into mature neurons, as well as NSC migration, by inhibiting the splicing and expression of mRNAs in NSCs. More recently, a nanodrug codelivering superparamagnetic iron oxide nanoparticles and small interfering RNA (siRNA)/antisense oligonucleotides targeting Pnky downregulated the levels of this lncRNA, therefore promoting the differentiation of NSCs into neurons and regeneration after cerebral stroke. Furthermore, this multifunctional nanodrug allowed in vivo tracking of NSCs with MRI visualization [180]. These findings indicate the great potential of lncRNA-based therapies in neuroregeneration in the future. Early targeting of fate-determining lnRNAs in the SVZ or other niches provides opportunities to boost endogenous neural regeneration.

### 4.8. Exosomes—Messengers and Directors

Exosomes are extracellular vesicles secreted by cells that transport cargos of proteins, lipids, and genetic material for cell-to-cell communication. Their cargo of bioactive molecules can modulate the cellular microenvironment and influence neurodegenerative disease progression. NSC-derived exosomes have recently emerged as compelling therapeutic candidates to combat neurodegenerative disease by targeting endogenous brain repair processes with no or minor side-effects. Reported mechanisms include: (1) neuroprotection—exosomes transferring microRNAs, transcription factors, and trophic factors that suppress neuronal apoptosis, inflammation, and oxidative damage [181] implicated in AD, PD, and ALS; (2) promoting neurogenesis—exosomal cargos include microRNAs and growth factors that stimulate neurogenesis [182]; and (3) pathological proteins clearance—transferring misfolded aggregated proteins to recipient cells with proteolytic capacity for degradation [183], including the amyloid-β and α-syn aggregates that drive AD and PD progression, respectively.

Optimizing the deployment of engineered therapeutic exosomes represents a niche-based medicine approach to harness endogenous NSCs and improve hostile microenvironments within neurodegenerative disease-affected tissues [22,140,184]. For example, they can be deployed to (1) activate resident NSCs, promoting their proliferation and differentiation into neural lineages via exosomal GF and morphogens; (2) mitigate toxic neuroinflammation via exosomal anti-inflammatory agents; or (3) provide trophic support and protective cues by delivering GF and cytokines to augment cell survival and regeneration.

However, there are several key challenges with using exosomes as a niche-based therapy for neurodegenerative diseases, including (1) standardization of exosome production and characterization protocols of therapeutic exosomes to ensure consistency and safety in clinical applications; (2) efficient noninvasive targeting of affected brain regions; and (3) navigating complex regulatory approval pathways for clinical testing.

## 5. From Niche Understanding to Deployment: Tools and Clinical Implications of Stem Cell Niche Dynamics

### 5.1. Direct In Vivo Reprograming: A Paradigm Shift in Cell Fate Regulation

Direct in vivo reprogramming, also known as transdifferentiation, is the process of directly altering the fate of fully differentiated cells within a living organism without them reverting to a pluripotent state. This approach has several advantages, such as eliminating the need for transplantation or ex vivo manipulation, avoiding potential immunological reactions, and ensuring controlled outcomes with reduced risk of tumorigenesis and mutations. Central to the success of in vivo reprogramming is the stem cell niche, which offers crucial environmental cues guiding the cell’s transformation. The niche’s intricate signaling and molecular milieu are essential in determining the appropriate transcription factors for targeted cell conversion. By leveraging insights from the niche, researchers can fine-tune the delivery of transcription factors, ensuring that the newly reprogrammed cells seamlessly integrate and operate as desired within the tissue [185,186,187].

### 5.2. Single-Cell Transcriptomics: Unraveling Niche Heterogeneity at Unprecedented Resolution

Single-cell transcriptomics allows for a comprehensive interrogation of the full RNA transcriptome produced by individual cells within complex tissues. This is particularly useful when studying stem cell niches, given their inherent cellular heterogeneity and multidimensional crosstalk among resident cells and communication with the environment. By analyzing the transcriptomes of individual cells within a niche, researchers can identify distinct cell types, understand their functional roles, gain profound insights into niche homeostasis, cell fate decisions, and potential perturbations in pathological states, and uncover the molecular signals that mediate interactions between stem cells and their niches [188,189,190,191].

### 5.3. 3D Organoids and Microfluidic Models

Organoids are three-dimensional cell aggregates or tissue structures derived from stem cells that mimic their original organs, thus preserving spatial information and enabling temporal change monitoring. These organoids largely replicate the cell composition and layout of the organs they come from, possibly even recreating a stem cell niche [17] and providing a controlled study environment. That allows for comprehensive experimentation and imaging of stem cells in their specific niches. Organoids made from purified cellular and extracellular elements facilitate direct modeling, the discovery of novel niche components, and the examination of stem cell–niche interactions. One of the main advantages is decoupling the signals within the niche from long-range signals. However, this also minimizes the understanding of how these signals interact with the outer environment.

Microfluidic chips enable precise recapitulation of stem cell microenvironments for mechanistic and therapeutic study. Lab-on-a-chip devices contain microscale fluid channels and chambers, allowing fine spatial and temporal control over cellular, chemical, and physical niche factors.

Neurodegenerative conditions like AD and Parkinson’s disease (PD) originate from dysfunction in neural stem cell niches. Microfluidics provides the potential to examine niche impairments closely. A recent “perivascular niche-on-a-chip” [16] used flow and 3D architecture to mirror the vascular niche, demonstrating maintenance of stemness. It also enabled analysis of cancer cell migration along microvessels, elucidating early steps in tumor propagation.

The stem cell niche presents myriad interacting elements—signaling/support cells, insoluble and soluble factors, matrix, metabolites, and biophysical cues. Microfluidics permits modular and dynamic niche reconstruction to determine the contribution of individual components. For example, microfluidic array devices allow high-throughput testing of how permutations of growth factors, matrix proteins, stiffness, and shear stress direct stem cell fate.

Microfluidic systems can also incorporate patient-specific cells for personalized testing. Microphysiological environments can identify variabilities in patient-derived NSCs that inform treatment. Furthermore, microfluidic multi-organ chips integrating neurovascular interfaces show the potential to screen patient niche therapy responses.

### 5.4. Neural Niche Dynamics in Disesases

#### 5.4.1. His and Hers: Sex Differences in Neurogenic Niches

**Figure 4:**
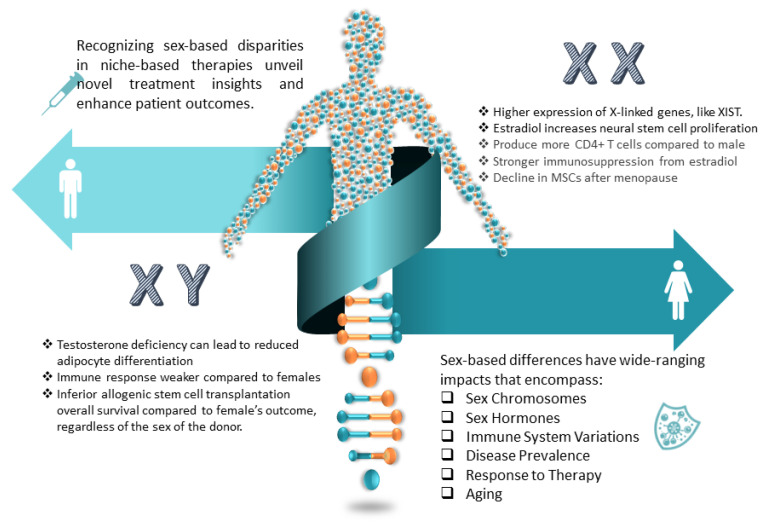
Sex-specific stem cell niches: understanding the unique biological mechanisms in men (XY) and women (XX) for enhanced neurodegenerative disease therapy.

It is becoming increasingly evident that biological sex plays a crucial role in the pathogenesis and progression of many neurodegenerative diseases (Figure 4). Promoting neurogenic potential by targeting dimorphic aspects of niche function optimizes treating neurodegeneration in men and women. Sex differences in neurogenic niche physiology provide plausible explanations for disparities in the age of onset, symptomatology, immune variations, disease course, and therapeutic responsiveness observed clinically in many neurological syndromes. For example, males with GBM have a higher incidence of worse outcomes [192] due to the upregulation of androgen receptors, estrogen’s protective role, the synergy of the immune system, hormones, and genetic and molecular differences. In [193,194], it was proved that stress induces lower adult neurogenesis in females, although it does not affect their working memory. Males’ new neurons have faster maturation and greater attrition rate compared to females [195], which may explain their better external stimuli neurogenesis response but inferior allogenic stem cell transplantation and survival rate [196,197]. Females have more robust immune responses [198], although estrogen, which promotes NSC proliferation and differentiation [199,200], also promotes stronger immunosuppression [201,202,203,204]. Due to females’ incomplete X inactivation and males’ hemizygosity for X-linked genes, potentially all brain cells show sex-based differences in gene expression [205]. Sex differences are reflected in aging, with stem cell declination in menopausal women [206,207,208,209].

#### 5.4.2. Aging: A Need for Rejuvenation

Adult NSCs in niche regions like the SVZ and DG supply new neurons involved in cognitive function. However, niche regenerative capacity declines with aging due to NSC depletion and microenvironmental changes. Understanding factors driving niche impairment has crucial implications for developing treatments for age-related neurodegenerative diseases. In adulthood, quiescent NSCs reside in vascularized niche sites until activated to proliferate and differentiate into neurons or glia. Aging disrupts the neurovascular environment and microglia function, negatively impacting NSC activation. Additionally, systemic changes impair existing NSC potency. This leads to reduced neuron production, contributing to cognitive decline and neurological disease susceptibility. Regenerative approaches aim to enhance endogenous NSC function for brain repair. Transplanted stem cells may replace damaged tissue, secrete proregenerative factors, or support NSCs directly. However, efficacy depends on aging and microenvironmental interactions [210,211,212,213,214].

#### 5.4.3. Translational Insights into Stem Cell Niche Disruptions in Neurodegenerative Diseases

The common factor across neurodegenerative diseases with respect to stem cell niches is that the disease pathology disrupts the local stem cell microenvironment and signals, impairing endogenous neurogenesis and the brain’s regenerative capacity. In other words, the endogenous stem cell niches lose their ability to self-renew, differentiate appropriately, and produce the types of neurons and glia needed by the brain. This impairs functional regeneration after neuronal loss.

Although the causes of many neurodegenerative diseases still need elucidation, the symptoms and anatomical and physiological changes can point to potential therapy pathways. For example, inflammation caused by disease (ALS, MS, AD, stroke, etc.) alters niche signaling and factors, reducing stem cell proliferation and differentiation. Besides inflammation altering niche signaling, other symptoms include the loss of neurons and glia, thus removing critical support cells; accumulation of toxic molecules interfering with niche signaling; disruption of niche vasculature; tumor growth distorting niches; and demyelination and loss of supraspinal connections, disconnecting niches from essential signals. Therefore, a key strategy is to restore niche homeostasis and signaling to re-mobilize neural stem cells in these diseases. This could involve clearing pathological factors, replacing lost cells, transplanting stem cells, delivering growth factors, or modulating inflammation through niche-based approaches. However, it is important to note that these are complex conditions, and the effectiveness of these strategies can vary greatly among individuals due to factors such as disease stage, individual health status, and genetic factors. Therefore, while the strategies are potential avenues of treatment, they are not guaranteed to be effective in all cases. Personalization of the therapy may be a key factor in therapy success.

Since the ultimate goal of niche-based therapy research is clinical translation, we are presenting two tables. In Table 3 are given implications for stem cell niche restoration based on the specific impacts of various neurodegenerative diseases: ALS, traumatic brain injury, stroke, multiple sclerosis (MS), spinal cord injury, spinal muscular atrophy (SMA), Lewy body dementia (LBD), glioblastoma (GBM), PD, and AD. In Table 4 are given examples of the niche-based therapies currently researched.

## 6. Niche Hacking: Breaking the Box

**Figure 5:**
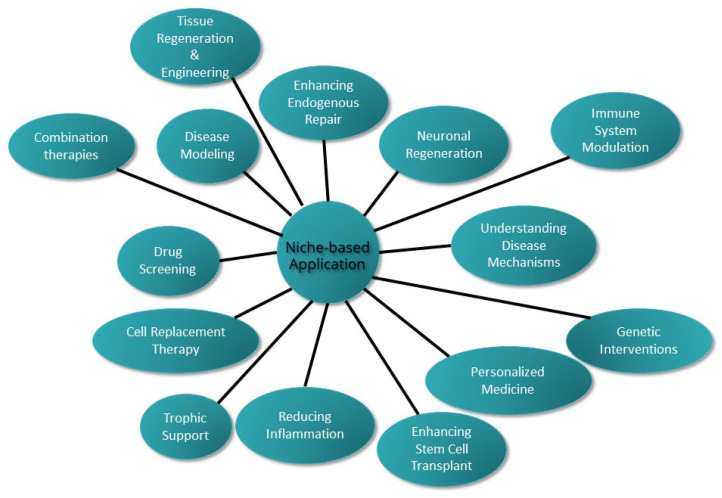
Niche-based deployment.

Summarizing the above text, we can easily conclude that NSC niches are dynamic, 3D environments in specialized anatomical locations with blurry borders and complex layers of intricate interacting networks of stem/progenitor cells, glia, blood vessels, neurotransmitters, and hormones, and that they change temporally and spatially based on internal and external cues. Alterations to interacting networks that impact neurogenic niches can compromise regenerative capacity and brain health. Niche-based approaches for neurodegenerative diseases have plenty of applications, some of which are presented in Figure 5. To design niche-based therapy, it is important to be aware of the current stage of the disease and the status of the niche. The time frame of the therapy administration may be crucial for the therapy success, i.e., for the therapy design and for selection of the best candidate for the therapy. For instance, the cerebrospinal fluid cytokine profiles in individuals with advanced autism spectrum disorder can be used as biomarkers to detect candidates who will not benefit from stem cell therapy [229]. The recent discovery of over 3000 novel neural cell types further expands the possibilities for cell therapy interventions, although differing complexity across brain regions presents integration challenges that will need to be addressed [230,231,232]. The newly discovered subarachnoid lymphatic-like membrane (SLYM) is a brain layer populated with myeloid cells that mediate inflammatory responses [182]. The SLYM’s strategic position allows it to surveil the CSF, and thus may facilitate interactions between intrathecally administered stem cells and endogenous brain niches. As the SLYM acts as an innate immune barrier, it would be interesting to investigate its role in transplanted stem cell homing and niches’ response to intrathecally transplanted cells.

### 6.1. System-of-Niches Models: A Synergistic Approach for Understanding Neurodegenerative Diseases

Many brain disorders are unique to humans, making it difficult to translate preclinical animal-based studies into clinical applications. That is due to physiological, anatomical, and biochemical differences between the species.

Organoids may be useful to bridge the interspecies differences gap. In addition, organoids may give personalized comprehension of the therapy, and personalized answers when designed from the patient’s cells, thus avoiding the perils of immunological reactions. Organoids can be useful for investigating the influence of exogenous miRNAs from plants, called botanmins [233]. It is noted that botanmins may modulate physiological and biochemical processes in humans via regulation of gene expression, although the exact mechanism needs elucidation. However, organoids alone have limitations in explaining disease mechanisms or niche functions due to topographic limitations and the failure to capture various developmental aspects accurately. To overcome those limitations, researchers introduced concepts of networking assembloids, i.e., region-specific organoids, or extended the deployment to the transplantation of human brain organoids in animals, and deployed optogenetic stimulations and microfluidics to improve the growth and functionality of the organoids [232,234,235,236,237,238]. Although these approaches may give insights with higher precision, several shortcomings may compromise the results; for example, the lack of external and long-distance connections of the niches with organs and body systems, or limited connections focused on a single axis, such as the gut–brain axis [239], in a single microfluidics setup. In the case of organoid transplants into animals, there is no clear distinction between the roles of animal- and human-specific molecules that influence stemness, such as ARHGAP11B [240,241]. Thus, the benefits of the hybrid environment may bring answers that are not natural to humans or are based on missing links.

A potential solution to these dilemmas is the creation of systems-of-niches and organs-on-a-chip, such as a hybrid of organoids, microfluidics, sensors, electromagnetic fields, in silico models, single-cell transcriptomics, direct in-organoid reprograming, and optogenetics, to mimic multiple system interactions, converging to the wholistic understanding of niche processes. For example, a system comprised of organoids generated from human induced pluripotent stem cells that can self-organize into various brain regions, including the neurogenic niches, and additional organoids to model other systems, such as, but not limited to, intestinal, liver, heart, and vascular organoids, to model the brain–organs connection. These organoids would be integrated into a microfluidic device that enables the precise control of biochemical and biophysical cues, interconnected through microfluidic channels lined with endothelial cells to facilitate inter-organ signaling, with media perfusing each component and independently controlled to enable the specific biochemical environment and modulate signaling. In addition, integrated sensors would monitor oxygen levels, shear stress, pressures, and nutrients and analyze circulating factors to replicate in vivo complexity closely. The system could incorporate microelectrodes or transparent regions for real-time monitoring of organoid development and function over time and in silico model analyses. The modularity also enables personalized or disease-specific models to be created. Since individual technology exists, this suggestion is feasible. Brain organoids alone and integrated with endothelial cell-lined microfluidic channels have been created to model the BBB, neuron–vascular interactions, and drug delivery to the brain. Separate microfluidic devices have housed intestinal, liver, cardiac, and other organoids. A dual gut–liver microfluidic chip that interconnected intestinal and liver organoids to model dietary effects and liver interactions, and a microfluidic BBB chip integrating vascular networks and neuro–glial culture that could be a building block for incorporating other organoids, exist. However, it is important to note that while this is a promising area of research, it is still in its early stages, and many technical challenges must be overcome, for instance, ensuring the survival and function of the organoids, replicating the complex cell–cell and cell–matrix interactions of the in vivo environment, and validating that the organoids accurately model the function of their in vivo counterparts. Nevertheless, the potential benefits for understanding human biology and disease, drug discovery, and regenerative medicine applications are enormous [239,242,243,244,245,246,247].

Computational models, named in silico to emphasize the silicon nature of computer parts, can integrate diverse data modalities from transcriptomics, proteomics, interactomics, and functional assays to map the molecular crosstalk governing NSC quiescence, activation, self-renewal, and differentiation. By revealing emergent properties of NSC regulation, modeling helps identify crucial hub signals and key regulatory motifs within niche interaction networks in healthy and diseased niches, and even predict therapeutic targets and biomarkers [248]. Machine learning further empowers prediction of optimal targets to manipulate niche-based stem cell activity [249]. Rapid expansion of artificial intelligence (AI) models has benefited the in silico models with a versatility in deployment scenarios, such as aiding the identification of stem cell components, the design of scaffolds, and imaging analyses to monitor the dynamics. Integration of in silico models with organoids and microfluidics further enhances their accuracy and relevance.

Early efforts have focused on integrating heterogeneous data modalities to reconstruct NSC regulatory networks. One of the main challenges is the integration of heterogeneous data sources, due to the diverse types of variables, mismatched distributions or scaling, and different data modalities [250]. For example, the Neurons–Glia–Vascular Interactions Nexus consolidates transcriptomic, proteomic, metabolomic, and interactomic data related to neuro–glia–vascular signaling [251]. By mapping connections between niche components, the platform identifies potentially targetable mechanisms that could enhance neurogenesis. However, many platforms lack spatial representations and mainly cover animal model data. Efforts to obtain high-quality human neurogenic niche data are crucial for clinical translation. Integrating models with electronic health record systems and trial data may also enable personalized predictions. Truly comprehensive niche models must bridge subcellular-, cellular-, tissue-, and system-level scales [252,253].

The hybrid or singular deployment of tools, such as in silico models, niches-on-chips, optogenetics, and quantum computing, can provide valuable insights into the behavior of neural stem cells and their interactions with their environment, cutting the costs of in vivo and in vitro research. However, it is important to note that while these models can provide valuable insights, they are still simplifications of the highly complex and dynamic in vivo environment. Therefore, results obtained from these models should be interpreted with caution and validated using in vivo experiments.

### 6.2. From Bench to Bedside: The Challenges in Translating

An integrated, systems-level niche perspective will likely be instrumental in realizing the full potential of regenerative therapy. However, translating these concepts into clinical reality involves overcoming numerous complex biological and technical obstacles, including: (1) precisely re-engineering niche dynamics and safely enhancing endogenous neurogenesis will require extensive optimization; (2) differences between model organisms with regenerative capacity and humans must be meticulously mapped; (3) long-term data are needed, as long-term consequences of niche alterations remain unclear; (4) clinically translating insights from basic science into safe, effective human applications is challenging; (5) identifying the critical niche components is complex since the niche involves multiple cell types, signals, and structures that vary by stem cell population; (6) comprehensive understanding of stemness differences in healthy and diseased niche; (7) avoiding off-target effects since niche-altering therapies could have unintended effects on other stem cell populations; (8) avoiding tumorigenesis and managing immune responses; (9) demonstrating functional recovery, which requires understanding the mechanisms and linking niche changes to measurable improvements in animal models or patients; and (10) establishing stable interventions, as developing niche-altering treatments that have a sustained, nontransient effect is challenging. Each of these challenges represents a significant area of focus in the field of regenerative therapy.

## 7. Deciphering Niche Complexity: The Core Link in Advancing Regenerative Medicine

Our understanding of the stem cell niche has progressed tremendously, yet many complexities remain unresolved. Moving forward, comprehensively integrating knowledge across niche aspects and achieving a systems-level perspective will be imperative. Elucidating dynamic interactions between cellular components, signaling pathways, epigenetic modifiers, electrical patterns, morphogens, immune mediators, and the ECM remains an ongoing challenge. Additionally, unknown niche locations, mechanisms, and cell types likely await discovery, and novel approaches, such as tissue nanotransfection, may open a technology to ad hoc niches [254].

Comparative studies of regeneration in highly capable species continue to provide evolutionary clues. However, translating these insights into human applications will require meticulous delineation of molecular differences. Novel techniques like biomaterials-based niche engineering and computational modeling will likely accelerate knowledge generation. In addition, hybrid approaches to combine several technologies may lead to new insights and overcome individual limitations. Some of the future deployments may include AI-driven quantum computing models, or exploration of quantum-based concepts to apply quantum phenomena to the neurogenic niche modulation based on quantum effects observed in biological systems.

Ultimately, the results of niche research must culminate in impactful clinical translation for neurodegenerative diseases. Comprehensive knowledge of niche components, interactions, spatial and temporal development, and dynamics in the healthy and diseased brain will enable targeted therapeutic strategies to safely reactivate endogenous neurogenesis and neural repair. This necessitates an intricate understanding of how niches change across the lifespan and in disease states. With this knowledge, we can develop targeted strategies to safely enhance endogenous regeneration that are rigorously optimized in preclinical models before thoughtful evaluation in human trials, or for re-evaluation of clinical trials [255].

Realizing the full promise of regenerative medicine requires the utmost respect for niche complexity. While daunting, embracing this vast intricacy through interdisciplinary collaboration offers immense hope. Harnessing the niche holds great promise for the future of regenerative therapies.

## Figures and Tables

**Table 1:** Comparison of conventional and niche-targeted treatment strategies for neurodegenerative diseases.

Approach	Conventional Strategies	Niche-Targeted Strategies
Goal	Manage symptoms and slow disease progression by targeting downstream effects	Stimulate neurogenesis and neural regeneration by targeting endogenous stem cells and niche factors [1]
Methods	Pharmacological agents, vitamins, lifestyle modifications	Growth factors, cytokines, gene and cell therapy, small molecules, biomaterials [1]
Targets	Neurotransmitter deficits, protein aggregation, inflammation, oxidative stress	Endogenous neural stem cells, support cells (astrocytes, endothelial cells), signaling factors (Wnt, BMP, Notch), vasculature, extracellular matrix (ECM) [9]
Mechanisms	Acetylcholinesterase inhibition (donepezil), NMDA receptor antagonism (memantine), dopamine replacement (levodopa), monoamine oxidase inhibition (selegiline), reactive oxygen species scavenging (vitamin E)	Niche pathway modulation, ECM modifications, anti-inflammatories, signaling molecule administration [9]
Outcomes	Modest symptom improvement, some modification of disease progression	Increased neurogenesis, integration of new neurons, possible reversal of cognitive/motor decline [9]
Limitations	Do not restore lost neurons or neural connections, benefits not sustained	Integrating new neurons, ensuring survival, unclear long-term benefits [10]

**Table 2:** List of the key regulatory functions of the stem cell niche.

Function	Description	Examples
Quiescence	The niche maintains stem cells in a nondividing quiescent state through factors like hypoxia, cell–cell contact, adhesive interactions, and signaling molecules. This preserves stemness and prevents premature exhaustion of the stem cell pool [11,12].	- Low oxygen levels in bone marrow niche - N-cadherin interactions in hematopoietic niche - Angiopoietin-1 induces quiescence in HSCs
Activation	The niche can rapidly activate quiescent stem cells in response to injury/stress signals, often mediated by inflammatory cytokines, growth factors, chemokines, ECM remodeling, and release of retention factors that trigger proliferation and mobilization [13,14].	- IFN-gamma, TNF-alpha induce HSC cycling - FGF2, EGF promote NSC activation after brain injury - SDF-1 gradient recruits circulating HSCs
Proliferation	The niche provides spatial and biochemical cues to ensure stem cells undergo symmetric, self-renewing cell divisions to maintain or expand the stem cell pool. This is regulated through signaling pathways like Wnt, Notch, Hedgehog, BMP [15,16,17,18,19,20,21].	- Wnt signaling sustains NSC proliferation - Notch signaling maintains neural progenitor pool - Shh promotes symmetric NSC division
Differentiation	The niche provides biochemical and biophysical cues to direct stem cell differentiation into specific lineages, often via morphogen gradients, ECM properties, epigenetic changes, and bioelectric signals [22].	- BMP, RA gradients specify differentiation - Soft vs. stiff ECM directs lineage - DNA methylation patterns alter gene expression - Ion channel expression influences differentiation
Homing	The niche produces chemotactic factors like SDF-1 to guide migration and homing of circulating stem cells back to their niche residence after injury or transplantation [23].	- SDF-1 expressed around brain lesions recruits NSCs
Engraftment	The niche provides adhesion molecules, ECM components, and growth factors to enable proper anchoring, survival, and integration of transplanted stem cells [12].	- Laminin, fibronectin aid stem cell engraftment - VEGF, BDNF support survival of transplanted cells
Immunomodulation	The niche shields stem cells from immune attack and modulates inflammatory signals through physical barriers, immunosuppressive molecules, and lack of MHC 1 on stem cells to foster a conducive environment [12].	- Tight junctions in SVZ limit immune access - TGF-beta inhibits T cell proliferation
Apoptosis	The niche tightly regulates stem cell numbers by inducing programmed cell death via death receptor signaling, cytokine withdrawal, or loss of survival signals to clear excess or dysfunctional stem cells [12].	- Growth factor removal triggers apoptosis

**Table 3:** Strategies and implications for restoring and regenerating stem cell niches in the context of various neurodegenerative diseases.

Condition	Impact Factor	Specific Impact on Disease	Implications for Stem Cell Niche Restoration and Regeneration
ALS [182]	Inflammation, loss of neurons and reactive glia	Alters signaling, reduces stemness.	Anti-inflammatory therapies, cell replacement strategies.
Traumatic Brain Injury [215]	Damaged neurons and glia	Impairs stemness and signaling.	Anti-inflammatory therapies, NSC transplantation, modulation of post-injury niche factors.
Stroke [216]	Disrupted niche vasculature, BBB integrity, epigenetic alterations	Reduces oxygen/nutrient supply, alters gene regulation.	Vascularization, transplantation of neural progenitors, epigenetic therapies.
MS [217]	Demyelination, inflammation	Disrupts signaling, alters cell communication.	GF, remyelinating agents, anti-inflammatory treatments.
Spinal Cord Injury [218]	Loss of niche factors and signals below the injury site reduces neurogenesis	Disconnects signaling, deprives of nutrients.	Delivering niche factors, molecules, and stem cell transplants may encourage regrowth and remyelination.
SMA [219]	Loss of neurons and glia, aging	Reduces support cells and signals, affects stem cell function with age.	NSC transplants, gene therapy, rejuvenation strategies.
LBD [220]	Toxic molecules—alpha-synuclein aggregation	Interferes with signaling pathways, disrupts neurogenic niches impairing dopamine neuron generation.	Molecule-clearing treatments with cell therapy to restore niches.
GBM [221]	Tumor growth, epigenetic alterations	Distorts niches, redirects stem cells to tumor growth, alters gene regulation.	Target tumor microenvironments to restrict tumorigenic niche signals, epigenetic modification therapies.
Parkinson’s [222]	Loss of neurons and glia, aging	Removes support cells and signals, aging affects stemness.	Cell replacement strategies, anti-aging therapies, GDNF, or other regenerative factors may encourage neurogenesis.
Alzheimer’s [223]	Toxic molecules, aging	Distorts niche signaling and neurogenesis.	Clearing toxic molecules, anti-inflammatory, rejuvenation and anti-aging strategies.

**Table 4:** Outcomes and limitations of niche modulations for cell therapies for various neurodegenerative disorders: research results.

Disease	Therapy	Relevant Outcomes	Limitations	Connection with Stem Cell Niche	Speculations on Interaction/Modulation of Niches
ALS [224,225]	Stem Cell Administration (MSC)	Slowed disease progression	Immune response to transplanted cells, limited long-term efficacy, variability in humans	Enhanced niche support	Transplanted stem cells may secrete factors that enhance the local niche environment, promoting neuronal survival and reducing neuroinflammation. This interaction can provide neuroprotection and support for damaged neurons.
	Exosomes	Neuroprotection	Purification	Modulation of niche signaling	Exosomes released by stem cells may contain bioactive molecules that can modulate niche signaling pathways, promoting neuroprotection and potentially reducing neuroinflammation.
Traumatic Brain Injury [215]	Stem Cell Administration	Improved cognitive function	Cell survival and integration	Niche-derived factors	Transplanted stem cells can interact with the local niche, receiving signals that promote their survival, differentiation, and integration into the injured tissue.
	mRNA	Enhanced neurorepair	Limited delivery to target site	Niche regulation of mRNA signaling	Stem-cell-derived mRNA therapies may be designed to respond to niche-specific signals, ensuring that they are activated in the appropriate microenvironment for neurorepair.
	MSC Exosomes	Improved neurological function, reduced inflammation in models	Nontargeted delivery, unclear mechanisms	Niche-derived exosomal factors	Exosomal bioactive molecules which can promote neuronal regeneration
Stroke [216,226]	Stem Cell Administration	Functional recovery	Immune rejection	Niche-mediated immune modulation	Transplanted stem cells can modulate the local immune response within the niche, reducing inflammation and promoting tissue repair.
	Modulating Wnt Signaling Pathways	Reduced neuroinflammation, neuroprotection, angiogenesis in ischemia models	Risk of tumorigenesis	Niche-related signaling pathways	Manipulating stem cell signaling pathways can indirectly affect the niche by altering the production of niche-related factors and reducing neuroinflammation.
Multiple Sclerosis [217,227,228]	Stem Cell Administration	Disease stabilization	Immune-related complications	Niche–immune interactions	Transplanted stem cells can interact with the immune cells in the niche, potentially modulating autoimmune responses and stabilizing the disease state.
	MSC-derived Exosomes	Neuroprotection	Standardization of exosome isolation, limited data	Niche-derived exosomal factors	Exosomes from stem cells may carry factors that can interact with and modulate the immune and glial cells within the niche, providing neuroprotection.
	mRNA	Enhanced axonal regeneration	Delivery challenges	Niche modulation of mRNA signaling	mRNA therapies can be designed to respond to niche-specific cues, facilitating axonal regeneration and remyelination.
Spinal Cord Injury [218]	Stem Cell Administration	Motor function improvement	Limited axonal regeneration	Niche support for axonal growth	Stem cells may interact with the niche to promote axonal regeneration by secreting factors that create a conducive microenvironment for axon growth and remyelination.
SMA [219]	Stem Cell Administration	Prolonged survival	Limited availability of suitable donors	Niche-mediated survival signals	Transplanted stem cells can receive signals from the niche that promote their survival and function, potentially prolonging patient survival.
LBD [220]	Stem Cell Administration	Improved cognitive function	Immune-related complications	Niche support for neural function	Transplanted stem cells may interact with the neural niche to enhance cognitive function through neuroprotection and synaptic support.
GBM [221]	Stem Cell Administration	Tumor suppression	Risk of promoting tumor growth	Niche influence on tumor microenvironment	Stem cells may interact with the tumor microenvironment, modulating it to suppress tumor growth and invasion.
Parkinson’s [222]	Stem Cell Administration	Improved motor function	Variable clinical responses	Niche support for dopamine production	Transplanted stem cells may enhance dopamine production within the niche, improving motor function in Parkinson’s disease patients.
Alzheimer’s [223]	Stem Cell Administration	Cognitive improvement	Limited engraftment in the brain	Niche support for cognitive function	Transplanted stem cells may enhance cognitive function by modulating the local niche environment and supporting neuronal health.
	Exosomes	Neuroprotection	Standardization of exosome isolation	Niche-derived exosomal factors	Exosomes released by stem cells may contain factors that interact with the niche to provide neuroprotection and support cognitive function.

## Data Availability

Not applicable.
